# Daratumumab May Attenuate Cardiac Dysfunction Related to Carfilzomib in Patients with Relapsed/Refractory Multiple Myeloma: A Prospective Study

**DOI:** 10.3390/cancers13205057

**Published:** 2021-10-09

**Authors:** Evangelos Terpos, Kimon Stamatelopoulos, Nikolaos Makris, Georgios Georgiopoulos, Ioannis Ntanasis-Stathopoulos, Maria Gavriatopoulou, Ageliki Laina, Evangelos Eleutherakis-Papaiakovou, Despina Fotiou, Nikolaos Kanellias, Panagiotis Malandrakis, Dimitris Delialis, Ioanna Andreadou, Efstathios Kastritis, Meletios A. Dimopoulos

**Affiliations:** 1 Department of Clinical Therapeutics, School of Medicine, National and Kapodistrian University of Athens, 11528 Athens, Greece; kstamatel@med.uoa.gr (K.S.); nickolasmak@hotmail.com (N.M.); ggeorgiop@med.uoa.gr (G.G.); johnntanasis@med.uoa.gr (I.N.-S.); mgavria@med.uoa.gr (M.G.); alaina@med.uoa.gr (A.L.); evelepapa@med.uoa.gr (E.E.-P.); desfotiou@med.uoa.gr (D.F.); nkanellias@med.uoa.gr (N.K.); panosmalan@med.uoa.gr (P.M.); ddelialis@med.uoa.gr (D.D.); ekastritis@med.uoa.gr (E.K.); mdimop@med.uoa.gr (M.A.D.); 2 Fondazione Toscana Gabriele Monasterio, 56124 Pisa, Italy; 3 Laboratory of Pharmacology, Faculty of Pharmacy, National and Kapodistrian University of Athens, 15771 Athens, Greece; jandread@pharm.uoa.gr

**Keywords:** carfilzomib, multiple myeloma, daratumumab, cardiovascular, heart, toxicity

## Abstract

**Simple Summary:**

The management of cardiovascular adverse events in patients with relapsed/refractory multiple myeloma undergoing treatment with carfilzomib can be challenging. Herein, we evaluated the potential cardioprotective effect of daratumumab when administered in combination with carfilzomib and dexamethasone (DaraKd). The study included 25 patients receiving either DaraKd (*n* = 14) or Kd (*n* = 11) who were evaluated for echocardiographic changes at the sixth cycle of treatment compared with baseline assessment. DaraKd was associated with preserved post-treatment cardiac systolic function compared with Kd. CD38 inhibition by daratumumab might restore metabolic disequilibrium in the cardiac tissue and prevent cardiac injury. A trend for a lower rate of cardiovascular adverse events among patients receiving DaraKd was also evident, although larger studies are needed to determine the association between echocardiographic and/or biomarker changes with cardiovascular adverse events.

**Abstract:**

Carfilzomib has improved survival in patients with relapsed/refractory multiple myeloma (RRMM), but it may exert cardiovascular adverse events (CVAEs). The aim of this study was to assess whether treatment with daratumumab may ameliorate carfilzomib-related toxicity. We prospectively evaluated 25 patients with RRMM who received either daratumumab in combination with carfilzomib and dexamethasone (DaraKd) (*n* = 14) or Kd (*n* = 11). Cardiac ultrasound was performed before treatment initiation and C6D16 or at the time of treatment interruption. Patients were followed for a median of 10 months for CVAEs. The mean (± SD) age was 67.8 ± 7.6 years and 60% were men. The two treatment groups did not significantly differ in baseline demographic characteristics (*p* > 0.1 for all). In the DaraKd group, we did not observe any significant change in markers of ventricular systolic function. However, these markers deteriorated in the Kd group; left ventricular (LV) ejection fraction, LV global longitudinal strain, tricuspid annular plane systolic excursion and RV free wall longitudinal strain significantly decreased from baseline to second visit (*p* < 0.05). A significant group interaction (*p* < 0.05) was observed for the abovementioned changes. CVAEs occurred more frequently in the Kd than the DaraKd group (45% vs. 28.6%). DaraKd was associated with preserved post-treatment cardiac systolic function and lower CVAE rate compared with Kd. The clinical significance and the underlying mechanisms merit further investigation.

## 1. Introduction

Although current treatment approaches in multiple myeloma (MM) have significantly enhanced patient outcomes, MM remains an incurable hematological malignancy. Proteasome inhibitors including bortezomib, carfilzomib and ixazomib are among the cornerstones of MM therapeutics [1,2]. Carfilzomib is a second-generation irreversible proteasome inhibitor that has been shown to improve overall survival (OS) in patients with relapsed and/or refractory multiple myeloma (RRMM) [3]. Carfilzomib has been approved either with dexamethasone (Kd) or with daratumumab and dexamethasonse (DaraKd) or with lenalidomide and dexamethasone (KRd) for patients for RRMM.

Carfilzomib may exert cardiovascular adverse events (CVAEs), although related mechanisms, prognostic markers and precipitating factors have not been fully characterized [4]. The CVAEs are more frequently reported in the first three months of treatment [5]. It has been estimated that patients with MM who receive carfilzomib may have an approximately 2-fold increased relative risk of heart failure [6].

Interestingly, in the prospective randomized phase 3 CANDOR study comparing DaraKd vs. Kd, a lower rate of cardiac failure events was observed in the DaraKd arm (7% vs. 10% with Kd) [7]. Importantly, new onset cardiac failure was the most common toxicity leading to carfilzomib discontinuation in both treatment groups (2% for both DaraKd and Kd) [7]. Daratumumab is a monoclonal antibody that targets CD38. Apart from myeloma cells, CD38 is expressed in several normal tissues including cardiomyocytes and immune cells of lymphatic origin and macrophages [8,9]. CD38 is highly expressed by endothelial cells, whereas CD38 expression mediates endothelial dysfunction induced by hypoxia-reoxygenation in the heart [10]. Pharmacological inhibition of CD38 exerts a cardioprotective effect by reducing endothelial damage post-ischemia [11,12]. In this context, the aim of this study was to assess whether treatment with daratumumab may attenuate carfilzomib-related cardiovascular toxicity.

## 2. Results

### 2.1. Baseline Characteristics 

The baseline characteristics of the study population and baseline echocardiographic markers are shown in Table 1. Eleven patients received Kd and 14 received DaraKd. The two treatment groups did not significantly differ in baseline demographic characteristics including age, gender and prevalence of hypertension, hyperlipidemia, smoking, diabetes mellitus and cardiovascular disease (*p* > 0.1 for all). In regard to echocardiographic characteristics, left ventricular (LV) mass, left atrial volume index (LAVi) and S wave of LV strain rate were higher whereas LV global longitudinal strain (GLS), peak atrial longitudinal strain (PALS) and right ventricular (RV) free wall longitudinal strain (LS) were lower in the Kd group.

### 2.2. Group Differences in Echocardiographic Changes after Treatment

#### 2.2.1. Markers of LV Systolic Function

**Figure 1:**
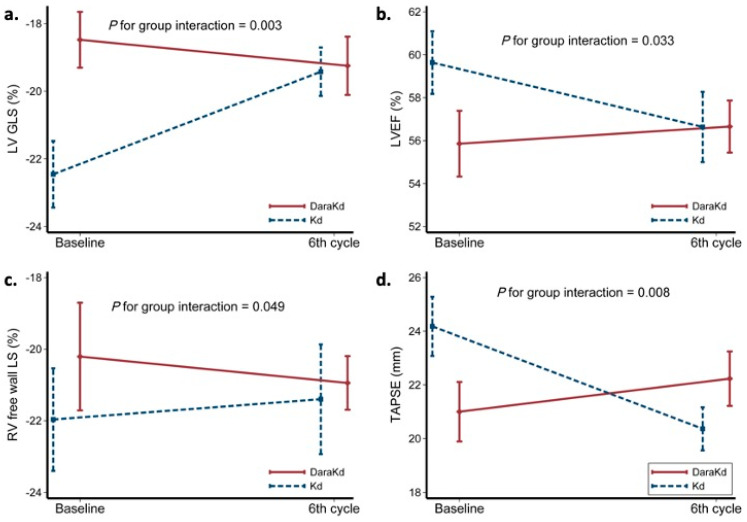
Line diagrams showing changes in LV GLS (**a**), LVEF (**b**), RV free wall strain (**c**) and TAPSE (**d**) and group interaction according to treatment assignment (Kd or DaraKd). *p* was derived from GLM ANCOVA.

As shown in Table 2, LVEF and LV GLS significantly decreased in the Kd but not in the DaraKd group. LV strain rate s wave decreased in both groups but reached significance only in DaraKd, while LV end-diastolic diameter (LVEDD) increased only in the DaraKdgroup. Specifically, in the Kd group, LVEF decreased from 59.6 ± 4.8 to 56.6 ± 5.4% (*p* = 0.026) and LV global longitudinal strain (GLS) from −22.5 ± 2.9 to −19.4 ± 2.1 (*p* = 0.007), whereas in the DaraKd group, LV strain rate s wave deteriorated from −1.38 ± 0.2 to −1.13 ± 0.1 s^−1^ (*p* = 0.005) and LVEDD increased from 46 ± 4.6 to 47.9 ± 3.8 mm (*p* = 0.047). By GLS ANOVA for repeated measurements there was a significant group interaction in the observed changes in LVEF and LVGLS, suggesting a less cardiotoxic effect of DaraKd compared with Kd (*p* < 0.05, Figure 1a,b). No other group interactions were observed in changes of LV systolic function markers.

#### 2.2.2. Markers of LV Diastolic Function

As shown in Table 2, LA diameter tended to increase while PALS and s wave of the LA strain rate tended to decrease in both groups. In particular, in the Kd group, LAVi increased from 40.95 ± 9.1 to 45.84 ± 8.8 mL/m^2^ (*p* = 0.002), PALS decreased from 28.7 ± 5.1 to 24.2 ± 5.6% (*p* = 0.030) and LA strain rate s wave deteriorated from 1.39 ± 0.18 to 1.14 ± 0.2 s^−1^ (*p* = 0.053), while in the DaraKd, LAVi increased from 31.45 ± 9.7 to 34.47 ± 10.8 mL/m^2^ (*p* = 0.003), PALS declined from 34.3 ± 5.4 to 29.1 ± 6.7% (*p* = 0.022) and LA strain rate s wave decreased from 1.92 ± 1.1 to 1.27 ± 0.6 s^−1^ (*p* = 0.102). No significant group interaction was observed for the reported changes in any of these markers.

#### 2.2.3. Markers of RV Systolic Function

TAPSE and RV free wall LS decreased only in the Kd group (Table 2). Specifically, TAPSE decreased from 24.18 ± 3.7 to 20.36 ± 2.7mm (*p* = 0.008) and RV free wall LS deteriorated from −31.9 ± 3.4 to −28.2 ± 4.3% (*p* = 0.012) in the Kd group, while, in the DaraKd group, TAPSE (21 ± 4.2 vs. 22.2 ± 3.7 mm, *p* = 0.438) and RV free wall longitudinal strain (−24.4 ± 4.3 vs. −25.8 ± 4.5%, *p* = 0.485) did not change significantly. A significant group interaction was observed for both parameters (Figure 1c,d).

### 2.3. Group Differences in CVAEs

Patients were closely followed for carfilzomib-related CVAEs, namely hypertension (HTN), heart failure (HF) and acute coronary syndrome (ACS). In a preliminary analysis, more events occurred in the Kd group (in 5 out of 11 patients, 45%, including 2 HF and 3 HTN grade 3 events) as compared with the DaraKd group (4 out of 14 patients, 28.6%, including 2 ACS, 1 HF and 2 HTN grade 3 events; one patient had two events, ACS and HTN, both of grade 3). An analysis of the association between changes in cardiac markers and CVAEs in the total population or by group was not possible because this study was designed for a mechanistic hypothesis and not for evaluating CVAEs related to changes in cardiac markers. This is the reason why changes in cardiac markers were, by design, not evaluated early before the occurrence of CVAEs. Indeed, the median time to CVAE was 12 days and all but two events occurred before the preset time point of follow-up echo study at sixth cycle of treatment. Thus, an association between changes and cardiac markers would not be interpretable. Furthermore, the study sample is limited to safely conduct a survival analysis.

## 3. Discussion

Carfilzomib-based regimens have been integrated in the treatment armamentarium and have significantly improved the outcomes of patients with MM [2,13,14,15]. However, cardiovascular complications associated with carfilzomib administration have become evident from both clinical trials and real-world studies. For this reason, consensus recommendations have been proposed for the early recognition and management of CVAEs related to carfilzomib [4,16]. A predictive model based on baseline office systolic blood pressure, 24 h blood pressure variability, left ventricular hypertrophy, pulse wave velocity and GLS has been recently proposed to predict the risk of CVAEs and stratify the patients in low and high-risk groups [17,18]. A baseline risk assessment based on traditional risk factors for CVAEs is also essential [4,16,19].

In the ENDEAVOR clinical trial, 11% (*n* = 51) of the 463 patients with RRMM, who received Kd with carfilzomib administered at 56 mg/m^2^ biweekly for 3 weeks on and 1 week off, experienced cardiac failure, whereas 3.9% (*n* = 18) presented with ischemic heart disease and 32.4% (*n* = 150) with hypertension [20,21]. In the ASPIRE clinical trial, 392 patients with RRMM received carfilzomib at 27 mg/m^2^ biweekly for 3 weeks on and 1 week off along with lenalidomide and dexamethasone. Among them, the incidence of cardiac failure was 6.4% (*n* = 25) and the incidence of ischemic heart disease was 5.9% (*n* = 23) [22]. A meta-analysis encompassing data from 24 prospective studies and 2594 patients with MM who received carfilzomib-based regimens showed that 18.1% (*n* = 617) experienced cardiovascular toxicities, whereas the pooled incidence of heart failure was estimated at 4.1% [23]. It has to be noted that higher doses of carfilzomib of at least 45 mg/m^2^ were associated with an increased rate of CVAEs, whereas a longer duration of infusion (30 min vs. 10 min) showed a marginal trend for CVAEs (*p* = 0.06) [23]. In another pooled analysis of 8 prospective studies, carfilzomib seemed to increase the risk of congestive heart failure but not the risk of ischemic heart disease in patients with RRMM [24]. More recently, in the CANDOR clinical trial, the incidence of cardiac failure was 7% (23/308) among patients with RRMM who received DaraKd and 10% (16/153) among those receiving Kd [7]. In a cross-study comparison between CANDOR and EQUULEUS studies, the administration of DaraKd with either biweekly carfilzomib at 56 mg/m^2^ or weekly carfilzomib at 70 mg/m^2^ did not have a significant difference in the incidence of cardiac failure grade 3 or greater (*n* = 2/185 or 1.1% vs. *n* = 2/85 or 2.4%, respectively) [25]. In the most recent meta-analysis including data on 5583 patients with MM from 45 prospective studies evaluating carfilzomib-based anti-myeloma regimens, the incidence of heart failure was estimated at 5.1% [26]. Interestingly, the risk for cardiotoxicity was not associated with any specific regimen (monotherapy vs. in combination with other agents) or with the disease setting (frontline vs. salvage treatment) [26].

In our study, we found that there was a significant group interaction in the observed changes in LVEF and LVGLS, which suggests a less cardiotoxic effect of DaraKd as compared with Kd. In the real-world setting, both LVEF and GLS have been reported to deteriorate following treatment with carfilzomib [27,28,29,30]. In a series of 62 patients with MM treated with carfilzomib, a decrease in LVEF was detected in 4 (6.5%) patients [30]. It has been shown that up to 12% (7/60) of the patients receiving carfilzomib may present with transient cardiac failure, as expressed by a reversible decrease in LVEF by at least 20% [27]. Interestingly, no association between the incidence of heart failure and the dose of carfilzomib or the duration of infusion was reported in a series of 60 patients [27]. LVEF has been traditionally considered as a marker of cardiotoxicity during cancer therapy [31], whereas GLS has emerged as a more sensitive marker that may predict early LVEF changes [32,33,34]. Importantly, a recent randomized clinical trial has shown that GLS-directed, prompt initiation of cardioprotective treatment in patients with cancer under cardiotoxic therapy can prevent a later decrease in LVEF [31,33]. A recent study on 88 myeloma patients under treatment with carfilzomib showed that changes in LV GLS are an early indicator of LV impairment [35]. In this context, LV GLS monitoring may become an early marker of carfilzomib-related cardiotoxicity that could guide therapeutic decisions even before a drop in LVEF becomes evident.

Furthermore, the addition of daratumumab in the Kd regimen did not reveal any significant effect on echocardiographic indices of LV diastolic function during treatment. A decline in LV diastolic markers has been also described in two series of patients with RRMM treated with carfilzomib [30,36]. Interestingly, early LV diastolic dysfunction may be associated with severe, carfilzomib-induced CVAEs that become evident later during the treatment course [36,37]. Thus, the assessment of LV diastolic function may enable the early intervention in order to prevent subsequent cardiovascular toxicity.

Regarding the evaluation of RV function, we found a deterioration in the echocardiographic indices of RV systolic function only in patients receiving Kd, which may indicate a protective effect of daratumumab. Two cases of severe right-sided heart failure with carfilzomib treatment have been also reported in the literature [38,39]. Anticancer therapy may lead to RV dysfunction similar to its adverse effects on LV function [40,41]. RV LS is a valuable marker for cardiac injury in patients with cancer under cardiotoxic treatment [42]. However, the prognostic significance of echocardiographic changes in RV function remains rather debatable [41,42,43]. In this context, the assessment of RV LS in patients with MM under carfilzomib-based treatment may serve as an early marker for RV cardiotoxicity in the clinical practice.

Carfilzomib-induced cardiotoxicity has been studied in preclinical models and is probably multifactorial. Carfilzomib is a potent and irreversible proteasome inhibitor. Inhibiting the proteasome-mediated degradation of misfolded metabolic products may result in a toxic effect for cardiac cells that may induce the autophagy of cardiomyocytes [44,45]. Additionally, proteasome inhibition may lead to increased reactive oxygen species and endothelial dysfunction, which has been associated with adverse cardiovascular events [46,47]. In cardiac tissue, carfilzomib inhibits the intracellular cascade of Akt/nitric oxide synthase which in turn results in decreased levels of nitric oxide that may predispose to cardiac dysfunction [48]. Carfilzomib increases PP2A that is a negative regulator of AMPKα/mTORC1 pathway and ultimately impairs the cardiac contractile activity [48]. Interestingly, the administration of metformin may reverse these carfilzomib-related cardiotoxic effects [48]. In a multi-omics integrative analysis, a downregulation of pyruvate along with an upregulation of lactate dehydrogenase B was detected in patients receiving carfilzomib who presented with CVAEs [49]. Furthermore, carfilzomib increases the expression of NF-κB, caspase-3, ERK and JNK, as well as the levels of malondialdehyde in cardiac cells, whereas it reduces the levels of cardiac glutathione and catalase enzyme activity [50]. A phosphodiesterase 4 inhibitor, apremilast, may reverse these molecular and enzymatic effects of carfilzomib in the cardiac tissue [50]. A flavonoid, rutin, may also attenuate the carfilzomib-induced cardiotoxic effects by restoring the oxidative/anti-oxidative homeostasis [51].

Since cardiomyocytes express CD38, the anti-CD38 monoclonal antibody daratumumab can regulate the CD38/cyclic adenosine diphosphate ribose/Ca^2+^ signaling pathway [52]. CD38 has emerged as a promising therapeutic target for cardio-protection as it has been implicated in the pathogenesis of several cardiovascular diseases including ischemia-reperfusion injury, atherosclerosis, cardiac arrhythmias, myocardial hypertrophy and pulmonary hypertension [53]. CD38 catalyzes the synthesis of nicotinic acid adenine dinucleotide phosphate (NAADP) and cyclic ADP-ribose (cADPR) from nicotinamide adenine dinucleotide (NAD) [54]. NAD is a key regulator of energy metabolism and is essential for vital cellular processes such as mitochondrial function [55]. The cardiac tissue is highly NAD-dependent due to its high mitochondrial load [55]. This is evident under hypoxia/reoxygenation conditions, which are characterized by CD38 upregulation that depletes NAD levels and in turn decreases nitric oxide and enhances reactive oxygen species (NOS) production [10]. Increased expression of CD38 by cardiac tissue-resident M1 macrophages has been also shown in immune-inflammatory conditions [56].

Furthermore, CD38 inhibits autophagic flux and the intracellular accumulation of autophagosomes, which is also induced by carfilzomib, ultimately results in cardiac dysfunction [57]. Interestingly, stimulation of beta-adrenoreceptor induces CD38-mediated NAD depletion [54,58]. NAADP and cADPR promote the development of heart failure by inducing cardiac hypertrophy, interstitial fibrosis and subsequent decrease in fractional shortening and ejection fraction [58]. Increased sympathetic activation due to both myeloma and active treatment may induce CD38 expression [59]. Preclinical studies have also shown that CD38 favors angiotensin II-induced cardiac hypertrophy [60]. Carfilzomib may promote the effect of angiotensin II on the cardiovascular system [61].

Pharmacological inhibition of CD38 by thiazoloquin(az)olin(on)e 78c or miR-499a-5p exerts a cardioprotective effect by reducing endothelial damage post-ischemia [11,12]. CD38 deficiency may result in reduced oxidative stress in the cardiac tissue, as well [62]. Daratumumab inhibits the cyclase activity of CD38, which regulates calcium release in the endoplasmic reticulum [52,63]. Clinical studies have shown that daratumumab may have a clinically insignificant impact on cardiac repolarization, which may be a result of its regulatory effect on calcium homeostasis in the cardiac tissue [64].

Taking all the above into consideration, daratumumab and carfilzomib might have opposite effects on NAD regulation, oxidative status and calcium homeostasis in cardiomyocytes. Therefore, daratumumab might counteract the contractile dysfunction induced by carfilzomib.

A limitation of our study pertains to the small number of patients in each treatment group, which reduces the strength of subgroup analyses. Furthermore, assessment of the association between changes in cardiac markers and CVAEs was possible by design. Larger studies are deemed essential in order to determine the potential cardioprotective effect of daratumumab in terms of CVAEs, as well as the role of monitoring echocardiographic indices and serum biomarkers such as NTproBNP in preventing CVAEs.

## 4. Materials and Methods

### 4.1. Study Design and Population

This is an ongoing prospective, single center, observational study conducted in the Department of Clinical Therapeutics of the National and Kapodistrian University of Athens (NKUA, Greece). Twenty-five patients with relapsed or refractory MM (RRMM) and eligible to receive carfilzomib-based treatment were enrolled. The inclusion and exclusion criteria are described in the Supplementary (Supplementary Material Table S1). All patients received the Kd regimen (carfilzomib 20/56 mg/m^2^ on days 1, 2, 8, 9, 15, 16 with dexamethasone 20 mg on these days, given in 28-day cycles). Patients in the DaraKd group also received daratumumab (at a weekly dose of 16 mg/kg, iv, for cycles 1–2, every 2 weeks for cycles 3–6 and every 4 weeks thereafter) until disease progression (PD), unacceptable toxicity of the treatment or withdrawal of consent.

All participants had a baseline visit, including medical history recording, cardiovascular risk factors assessment and detailed physical examination. During baseline visit, all patients had an echocardiographic study, which was repeated at 6 months following drug initiation or earlier if treatment interruption was indicated. One patient (from the DaraKd group) denied follow-up. In 2 patients from the Kd group only standard echocardiographic measurements were used in the analysis due to inadequate quality for speckle tracking analysis. For the same reason, RV strain measurements in 1 patient from the DaraKd group and LA strain measurements in 1 patient from the Kd group were not analyzed. Patients were closely followed for carfilzomib-related CVAEs, namely HTN, HF and ACS.

The primary objective of the study was to evaluate differences in post-treatment changes in echocardiographic markers in response to DaraKd vs. Kd. The study was approved by the Local Ethics Committee of Alexandra Hospital (approval reference number 396/12-05-2017) and was conducted in full compliance with HIPAA and the principles of Good Clinical Practice and the Declaration of Helsinki. All patients provided written informed consent.

### 4.2. Standard Echocardiography

At baseline, patients underwent a transthoracic echocardiography including standard echocardiographic images and specific images appropriate for speckle tracking processing. On day 16 of cycle 6 (C6D16) a follow-up echocardiographic study was performed based on the aforementioned protocol. Echocardiographic studies were performed by a single experienced operator using a standard commercial echocardiographic system (Vivid 7; GE Medical Systems, Milwaukee, WI, USA).

Standard echocardiographic images were acquired according to the recommendations of the European and the American Associations of Echocardiography [65]. Left ventricular ejection fraction (LVEF) and left atrial volume (LAV) were estimated using the biplane method and the latter was indexed (LAVi) with body surface area (BSA). Early diastolic (Ea) mitral annular velocity was calculated as the average of septal and lateral mitral annular velocities. LV diastolic dysfunction (LVDD) grade was calculated based on current recommendations [66].

### 4.3. Speckle Tracking Parameters

Echocardiographic images were processed using commercially available 2D speckle tracking software (EchoPAC PC version 204; GE Medical Systems, Milwaukee, WI, USA) according to published guidelines [67,68]. Speckle tracking parameters were calculated for the left ventricle (LV), right ventricle (RV) and left atrium (LA). Global LV longitudinal strain (GLS), and average LV longitudinal strain rate S and E waves were calculated from the three apical views. LV radial strain was calculated at the level of the papillary muscles. LA strain parameters were estimated by averaging 4-chamber and 2-chamber apical views. Peak atrial longitudinal strain (PALS) was defined as the peak strain at reservoir function. Intra-observer variability was assessed in 10 random MM patients of our cohort and 7 healthy individuals from the staff of our hospital. Intraclass correlation coefficients, calculated between 2 successive measurements a few days apart, were excellent (>0.9) for all the markers evaluated. All measurements were performed by the same person. ICC values for each marker are shown in Supplementary Material Table S2. The reference values for healthy individuals are as follows: GLS −22.5 ± 2.7%, radial 39.2 ± 9.9% [69], LV strain rate 1.34 ± 0.27 s^−1^ [70], PALS 35.7 ± 5.8%, LA strain rate 1.43 ± 0.24 s^−1^ [71], RV strain 24.5 + 3.8% and RV free wall strain 28.5 + 4.8% [72].

### 4.4. Follow-Up and Adjudication of Cardiovascular Adverse Events (CVAEs)

Patients were followed for a median of 10 months for carfilzomib-related CVAEs (HTN, HF, ACS). Clinical evaluation was performed by two trained physicians and a cardiologist. Response to treatment and disease progression were defined according to the International Myeloma Working Group criteria [73]. All adverse events were graded according to the US National Cancer Institute Common Terminology Criteria for Adverse Events v4.03.

### 4.5. Statistical Methods

Continuous variables are presented as mean ± standard deviation (SD) or median (interquartile range) values. Nominal variables are summarized as counts and valid percentages. Histograms were used to assess the distribution of continuous variables. Baseline differences between the treatment groups were evaluated by the two independent samples Student’s *t*-test and the Mann–Whitney U Test for continuous variables or the chi-squared test for nominal variables. Next, we employed analysis of variance (ANOVA) for repeated measures and assessed differential changes in cardiac markers of interest for the two treatment groups across the follow-up. We performed comparisons in echo indices pre- and post-treatment within each treatment group separately. We specifically examined the interaction term between treatments (DaraKd vs. Kd) and time (end of follow up vs. baseline) in ANOVA for repeated measurements models to adjudicate a treatment-specific effect on cardiac markers across cycles of therapy (“within-between effect”). More specifically, we did not focus either on changes within-group (i.e., the difference between follow-up and baseline for echocardiography variable separately in Kd group or DaraKD group) or on differences between-group (i.e., the difference between Kd group or DaraKD group and baseline) but tested the interaction between grouping (Kd vs. DaraKD) and changes of the dependent variable across the study period. Importantly, this flexible and robust statistical technique takes into consideration both measurements (baseline and follow up) and infers whether the trajectory of the dependent variable for each group differs (parallel or deviating lines) between the two populations (with or without Dara treatment) [74]. Given the small number of patients per group, which did not allow for complete randomization of treatment allocation, it is not surprising that certain echocardiography attributes differ between the two groups at baseline; still, our main statistical inference is based on the interaction of between-subjects factor (group) with the within-subjects term (longitudinal changes in echo variables) which controls for baseline differences and the correlation of measurements for each participant. Results for this statistical test can be found under the last column of Table 2. In the same Table, we have also provided within-group fluctuations in echocardiography variables for completeness and to facilitate understanding of the direction and magnitude of changes. The level of statistical significance was pre-specified at *p* < 0.05. All tests were two-tailed. We used all available data post-inclusion in this pilot analysis and no formal power calculations were performed. Statistical analysis was conducted with IBM SPSS Statistics v23 (IBM, Armonk, NY, USA).

## 5. Conclusions

In conclusion, our study suggests that daratumumab in combination with carfilzomib-based treatment for RRMM is beneficial by attenuating carfilzomib-induced echocardiographic changes regarding both LV and RV function. Although the underlying pathophysiology of the potential cardioprotective effect of daratumumab remains to be elucidated, our findings have important clinical implications for the cardiac monitoring of patients receiving carfilzomib-based anti-myeloma regimens.

## Figures and Tables

**Table 1:** Baseline patient characteristics.

Baseline Parameters	Kd (*n* = 11)	DaraKd (*n* = 14)	*p* Value
Age (years), mean ± SD	69.45 ± 9	66.43 ± 6.3	0.34
Male gender, [*n* (%)]	7 (50)	8 (72.7)	0.26
Prior lines of therapy (1 vs. 2)	6 vs. 5	11 vs. 3	0.20
Prior HDM/ASCT	1	3	0.40
Prior PI	10	13	0.86
Prior IMiD	8	12	0.42
BMI (kg/m^2^), mean ± SD	28.91 ± 2.6	28.89 ± 3.8	0.98
Hyperlipidemia, [*n* (%)]	0 (0)	3 (21.4)	0.1
Smoking, [*n* (%)]	2 (18.2)	2 (14.3)	0.79
Cardiovascular disease, [*n* (%)]	4 (36.4)	4 (28.6)	0.68
Hypertension, [*n* (%)]	6 (54.5)	6 (42.9)	0.56
Diabetes, [*n* (%)]	2 (18.2)	1 (7.1)	0.4
Kidney disease, [*n* (%)]	1 (9.1)	0 (0)	0.25
Systolic BP (mmHg), mean ± SD	138.09 ± 13.4	126.7 ± 21.1	0.13
Diastolic BP (mmHg), mean ± SD	71.64 ± 9.2	68.64 ± 7.9	0.39
**Echocardiographic Parameters**			
LV diastolic diameter (mm), mean ± SD	50.14 ± 5.8	46 ± 4.6	0.58
LV mass (g), mean ± SD	178.4 ±49.7	125.9 ± 30.3	0.003
LVEF (%), mean ± SD	59.64 ± 4.8	55.86 ± 5.7	0.094
LV GLS (%), mean ± SD	−22.46 ± 2.9	−18.48 ± 3	0.006
LV strain rate s wave (s^−1^), mean ± SD	−1.16 ± 0.3	−1.38 ± 0.2	0.071
LV Radial strain (%), mean ± SD	52.56 ± 22.7	46.51 ± 22	0.545
Diastolic dysfunction, [*n* (%)]	10 (90.9)	10 (71.4)	0.23
E/Ea, mean ± SD	11.18 ± 4.5	9.64 ± 3.6	0.35
LA diameter (mm), mean ± SD	43.6 ± 7.6	34.7 ± 4.6	0.037
LAVi (mL/m^2^), mean ± SD	40.95 ± 9.1	31.45 ± 9.7	0.032
PALS (%), mean ± SD	28.72 ± 5.1	34.34 ± 5.4	0.029
LA strain rate s wave (s^−1^), mean ± SD	1.39 ± 0.18	1.92 ± 1.1	0.66
RVSTDI (cm/s), mean ± SD	13.91 ± 2.6	13.31 ± 2.4	0.56
TAPSE (mm), mean ± SD	24.18 ± 3.7	21 ± 4.2	0.1
RV GLS (%), mean ± SD	−21.97 ± 4.3	−20.21 ± 5.2	0.42
RV free wall LS (%), mean ± SD	−31.89 ± 3.4	−24.39 ± 4.3	<0.001

BMI: body mass index; BP: blood pressure; LVEF: left ventricular ejection fraction; LV: left ventricular; LA: left atrial; E/Ea: ratio of early diastolic transmitral flow velocity to early diastolic mitral annulus velocity; IVS: interventricular septum; RV: right ventricular; RVSTDI: systolic tricuspid annulus velocity; TAPSE: tricuspid annulus plain systolic excursion; GLS: global longitudinal strain; LAVi: left atrial volume index; PALS: peak atrial longitudinal strain; LS: longitudinal strain; HDM/ASCT: high dose melphalan/autologous stem cell transplant; PI: proteasome inhibitor; IMiD: immunomodulatory drug.

**Table 2:** Changes in echocardiographic markers according to treatment assignment.

Treatment	Echocardiographic Parameter	Baseline Values (Mean ± SD)	Follow-Up Values (Mean ± SD)	*p* Value	*p* for GroupInteraction
**LV function**					
Kd	LVEF (%)	59.64 ± 4.8	56.64 ± 5.4	0.026	**0.033**
DaraKd		55.86 ± 5.7	56.65 ± 4.4	0.539	
Kd	LV diastolic diameter (mm)	50.14 ± 5.8	50.23 ± 6.3	0.898	0.179
DaraKd		46 ± 4.6	47.85 ± 3.8	0.047	
Kd	LV GLS (%)	−22.46 ± 2.9	−19.42 ± 2.1	0.007	**0.003**
DaraKd		−18.48 ± 3	−19.25 ± 3.1	0.309	
Kd	LV strain rate s wave (s^−1^)	−1.16 ± 0.3	−1.09 ± 0.2	0.383	0.111
DaraKd		−1.38 ± 0.2	−1.13 ± 0.1	0.005	
Kd	LV radial strain (%)	52.56 ± 22.7	49.56 ± 26.2	0.589	0.818
DaraKd		46.51 ± 22	41.39 ± 12.4	0.463	
Kd	LAVi (mL/m^2^)	40.95 ± 9.1	45.84 ± 8.8	0.002	0.182
DaraKd		31.45 ± 9.7	34.47 ± 10.8	0.003	
Kd	E/Ea	11.18 ± 4.5	11.73 ± 6	0.512	0.919
DaraKd		9.64 ± 3.6	10 ± 3	0.547	
Kd	PALS (%)	28.72 ± 5.1	24.22 ± 5.6	0.030	0.808
DaraKd		34.34 ± 5.4	29.14 ± 6.7	0.022	
Kd	LA strain rate s wave (s^−1^)	1.39 ± 0.18	1.14 ± 0.2	0.053	0.540
DaraKd		1.92 ± 1.1	1.27 ± 0.6	0.102	
**RV function**					
Kd	TAPSE (mm)	24.18 ± 3.7	20.36 ± 2.7	0.008	**0.008**
DaraKd		21 ± 4.2	22.23 ± 3.7	0.438	
Kd	RVSTDI (cm/s)	13.91 ± 2.6	14 ± 2.3	0.911	0.347
DaraKd		13.31 ± 2.4	12.54 ± 1.8	0.137	
Kd	RV LS	−21.97 ± 4.3	−21.4 ± 4.6	0.547	0.583
DaraKd		−20.21 ± 5.2	−20.95 ± 2.6	0.705	
Kd	RV free wall LS (%)	−31.89 ± 3.4	−22.23 ± 4.3	0.012	**0.049**
DaraKd		−24.39 ± 4.3	−25.75 ± 4.5	0.485	

LVEF: left ventricular ejection fraction; LV: left ventricular; LA: left atrial; LAVi: left atrial volume index; E/Ea: ratio of early diastolic transmitral flow velocity to early diastolic mitral annulus velocity; IVS: interventricular septum; RV: right ventricular; RVSTDI: systolic tricuspid annulus velocity; TAPSE: tricuspid annulus plain systolic excursion; GLS: global longitudinal strain; PALS: peak atrial longitudinal strain; LS: longitudinal strain; Bold values denote statistical significance.

## Data Availability

Data available upon request from the corresponding author.

## Supplementary Materials

The following are available online at www.mdpi.com/article/10.3390/cancers13205057/s1, Table S1: Inclusion and exclusion criteria; Table S2: ICC Values.
