# Supplementary material for: Daratumumab May Attenuate Cardiac Dysfunction Related to Carfilzomib in Patients with Relapsed/Refractory Multiple Myeloma: A Prospective Study
Source: Cancers (Basel). 2021 Oct 9;13(20):5057. doi: 10.3390/cancers13205057 (PMC8533991; doi:10.3390/cancers13205057)
Supplement: Supplementary file 1 [file cancers-13-05057-s001.zip › cancers-1399845-supplementary-done.pdf]

Supplementary material

Daratumumab May Attenuate Cardiac Dysfunction Related to Carfilzomib in Patients with Relapsed/Refractory Multiple Myeloma; A Prospective Study

Table S1. Inclusion and exclusion criteria.

**Inclusion criteria**
Males and females at least 18 years of age
Voluntary written informed consent before performance of any study-related procedure
Documented relapsed or refractory multiple myeloma in need of therapy, after at least one previous line of therapy for myeloma
Eastern Cooperative Oncology Group (ECOG) performance status score of ≤ 2
Willingness and ability to participate in study procedures
**Exclusion criteria**
Anti-myeloma treatment within 2 weeks prior to Cycle 1, Day 1
Cumulative dose of corticosteroids greater than or equal to the equivalent of 140mg prednisone for ≥4 days or a dose of corticosteroids greater than or equal to the equivalent of 40 mg/day of dexamethasone for ≥4 days within the 2-week period prior to Cycle 1, Day 1
Clinically significant cardiac disease, including:<br>Myocardial infarction within 6 months, or unstable or uncontrolled condition (e.g., unstable angina, congestive heart failure, New York Heart Association Class III-IV)<br>Cardiac arrhythmia (CTCAE Grade 2 or higher) or clinically significant ECG abnormalities<br>ECG showing a baseline QT interval as corrected by Fridericia's formula (QTcF) >470 msec
Known active HBV, HCV or HIV.
Any of the following laboratory test results at baseline:<br>Absolute neutrophil count ≤1.0 × 10^9/L<br>Hemoglobin level ≤7.5 g/dL (≤5 mmol/L)<br>Platelet count <75 × 10^9/L (<50x10^9/L in case of more than 50% bone marrow plasma cell invasion)<br>Alanine aminotransferase level ≥2.5 times the upper limit of normal
Pregnant or nursing women

Table S2. ICC Values.

Parameter	ICC Value for Healthy Individuals	ICC Value for Multiple Myeloma Patients
LVEF	0.934	0.964
LV GLS	0.990	0.995
LV strain rate S wave	0.994	0.985
LV strain rate E wave	0.973	0.983
LV Radial strain	0.952	0.991
PALS	0.992	0.990
LA strain rate S	0.980	0.992
RV free wall strain	0.943	0.939
RV strain	0.943	0.913

LVEF; left ventricular ejection fraction, LV; left ventricular, LA; left atrial, RV; right ventricular, GLS; global longitudinal strain, PALS; peak atrial longitudinal strain.
